# A 9-aminoacridine derivative induces growth inhibition of Ehrlich ascites carcinoma cells and antinociceptive effect in mice

**DOI:** 10.3389/fphar.2022.963736

**Published:** 2022-10-17

**Authors:** Vivianne M. Mangueira, Tatyanna K. G. de Sousa, Tatianne M. Batista, Renata A. de Abrantes, Ana Paula G. Moura, Rafael C. Ferreira, Reinaldo N. de Almeida, Renan M. Braga, Fagner Carvalho Leite, Karina C. de P. Medeiros, Misael Azevedo T. Cavalcanti, Ricardo O. Moura, Geovana F. G. Silvestre, Leônia M. Batista, Marianna V. Sobral

**Affiliations:** ^1^ Post Graduation Program in Bioactive Natural and Synthetic Products, Federal University of Paraíba, João Pessoa, Brazil; ^2^ Department of Morphology, Federal University of Rio Grande do Norte, Natal, Brazil; ^3^ Drug Development and Synthesis Laboratory, Department of Pharmacy, State University of Paraíba, João Pessoa, Brazil; ^4^ Department of Pharmaceutical Sciences, Federal University of Paraíba, João Pessoa, Brazil

**Keywords:** acridine derivatives, Ehrlich tumor, toxicity, immunomodulation, antiangiogenic action, antinociceptive activity, opioid pathway

## Abstract

Acridine derivatives have been found with anticancer and antinociceptive activities. Herein, we aimed to evaluate the toxicological, antitumor, and antinociceptive actions of N’-(6-chloro-2-methoxyacridin-9-yl)-2-cyanoacetohydrazide (ACS-AZ), a 9-aminoacridine derivative with antimalarial activity. The toxicity was assessed by acute toxicity and micronucleus tests in mice. The *in vivo* antitumor effect of ACS-AZ (12.5, 25, or 50 mg/kg, intraperitoneally, i.p.) was determined using the Ehrlich tumor model, and toxicity. The antinociceptive efficacy of the compound (50 mg/kg, i.p.) was investigated using formalin and hot plate assays in mice. The role of the opioid system was also investigated. In the acute toxicity test, the LD_50_ (lethal dose 50%) value was 500 mg/kg (i.p.), and no detectable genotoxic effect was observed. After a 7-day treatment, ACS-AZ significantly (*p* < 0.05) reduced tumor cell viability and peritumoral microvessels density, suggesting antiangiogenic action. In addition, ACS-AZ reduced (*p* < 0.05) IL-1β and CCL-2 levels, which may be related to the antiangiogenic effect, while increasing (*p* < 0.05) TNF-α and IL-4 levels, which are related to its direct cytotoxicity. ACS-AZ also decreased (*p* < 0.05) oxidative stress and nitric oxide (NO) levels, both of which are crucial mediators in cancer known for their angiogenic action. Moreover, weak toxicological effects were recorded after a 7-day treatment (biochemical, hematological, and histological parameters). Concerning antinociceptive activity, ACS-AZ was effective on hotplate and formalin (early and late phases) tests (*p* < 0.05), characteristic of analgesic agents with central action. Through pretreatment with the non-selective (naloxone) and μ1-selective (naloxonazine) opioid antagonists, we observed that the antinociceptive effect of ACS-AZ is mediated mainly by μ1-opioid receptors (*p* < 0.05). In conclusion, ACS-AZ has low toxicity and antitumoral activity related to cytotoxic and antiangiogenic actions that involve the modulation of reactive oxygen species, NO, and cytokine levels, in addition to antinociceptive properties involving the opioid system.

## 1 Introduction

Cancer is a prominent pathology worldwide, and its treatment remains a challenge, mainly due to its toxicity and chemoresistance. According to the World Health Organization, in the year 2020, about 10 million deaths were attributed to cancer (World Health Organization, 2022).

The cancer progression depends on the induction of angiogenesis and the formation of an inflammatory tumor microenvironment, allowing tumor growth and development and its metastatic spread (Hanahan and Weinberg, 2011; Hanahan, 2014; Manasa and Kannan, 2017; Tapia-Vieyra et al., 2017). In this sense, angiogenesis and tumor inflammation as hallmarks of câncer have gained prominence in the search for therapies that modulate these processes.

Tumor angiogenesis is associated with the overexpression of pro-angiogenic factors, such as some cytokines (Lugano et al., 2020; Teleanu et al., 2020), reactive oxygen species (ROS) (Huang and Nan, 2019), and nitric oxide (NO) (Miranda et al., 2021). Angiogenesis provides the tumor with nutrients, oxygen, and a means of waste removal; thus antiangiogenic therapies are potential cancer treatments. We can highlight the essential role of cytokines in tumor inflammation related not only to angiogenesis but also to tumor development, survival, and metastasis (Hinshaw and Shevde, 2011; Mao et al., 2021). In this context, cytokines can drive the antitumor immune response. However, they can also act as pro-tumorigenic factors by attracting immune cells that contribute to tumor development (Naito et al., 2017; Oura et al., 2021; Ozga et al., 2021). Therefore, the modulation of cytokines in the tumor by potential anticancer drugs has attracted much attention and has been suggested as a target for cancer therapy.

The challenge of developing more effective and less toxic drugs has stimulated the design of several scaffolds for cancer treatment. In this sense, the acridine scaffold is widely explored as a potentially scaffold for discovering novel anticancer drugs due to its ability to intercalate into DNA (Belmont and Dorange, 2008; Rupar et al., 2020). Acridines are heterocycles that consist of two rings fused to a centrally positioned pyridine ring. These compounds have a planar structure that allows them to interfere with a variety of metabolic processes, including insertion between DNA base pairs, inhibition of topoisomerases, telomerase, and protein kinases. In this way, they promote different biological activities (Gouveia et al., 2018; Silva et al., 2018), such as antibacterial, antimalarial, antiprotozoal, antifungal, antitrypanosomal, antileishmanial, and anticancer activity (Chen et al., 2014).

Amsacrine is an anticancer acridine derivative used in the treatment of leukemia and lymphoma. It was the first synthetic topoisomerase II poison approved for clinical use (Rupar et al., 2020). Literature reports have shown the effects of 9-aminoacridine derivatives, based on amsacrine scaffold, as potential anticancer drugs (Belmont and Dorange, 2008; Kozurkova et al., 2020; Kumar et al., 2013; Rupar et al., 2020; El-Sayed et al., 2022). Additionally, the mechanism of action for 9-aminoacridine-based anticancer drugs involves cell cycle arrest and apoptosis induction (Sun et al., 2015), and effects on the PI3K/AKT/mTOR, NF-κB and p53 signaling pathways (Guo et al., 2008; Guo et al., 2009).

We have previously reported two spiro-acridine derivatives, a thiophene–acridine hybrid, and a 9-aminoacidrine derivative as potential anticancer agents (Duarte et al., 2020; Lisboa et al., 2020; Mangueira et al., 2017; Silva et al., 2019). The anticancer potential of the spiro-acridine compounds was associated with antiangiogenic action and up-regulation of Th1-type responses, mainly by inducing the increase of TNF-α and IL-1β levels in the tumor microenvironment (Duarte et al., 2020; Silva et al., 2020). We also published the synthesis and antitumor effect of a new 9-aminoacridine derivative by inducing cell cycle arrest and angiogenesis (Mangueira et al., 2017). Although there is much scientific interest in these compounds, there is very little information regarding the correlation between the modulation of crucial factors in the tumor microenvironment (such as cytokines, ROS, and NO) and the cytotoxicity and inhibition of angiogenesis by 9-aminoacridine derivatives.

**FIGURE 1:**
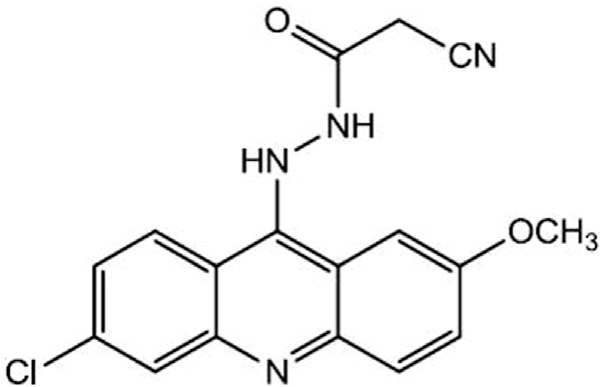
Chemical structure of acridine derivative ACS-AZ.

In a continuation of the research interest in novel acridine derivatives, it was synthesized the N’-(6-chloro-2-metoxyacridin-9-yl)-2-cyanoacetohydrazide (ACS-AZ) (Figure 1), a 9-aminoacridine derivative which showed antimalarial activity (Silva et al., 2018). Literature reports have shown that antimalarial medicines may also display anticancer properties, including circumventing multi-drug resistance (Ellis et al., 2021). Therefore, also considering the strong basis to further explore the potential application of this antimalarial compound in cancer, we evaluated its antitumor action on the Ehrlich ascites carcinoma model.

In addition to the activity against microorganisms and tumor cells, the analgesic property has also been described for acridine derivatives (Llama et al., 1989; Sondhi et al., 2004; Chandra et al., 2010). However, there is no data in the literature related to their mechanisms of antinociceptive action. Pain is a subjective and common experience with sensory-discriminative, affective-motivational, and cognitive-evaluative elements (Lindsay et al., 2021). It has been estimated that pain can affect about 30% of the world population (Cohen et al., 2021); thus, the high prevalence of people, especially with chronic diseases, such as arthritis, fibromyalgia, and cancer, across the globe has made pain management come to the forefront of the public health community (Orhurhu et al., 2021). Pharmacologic management of pain includes nonsteroidal anti-inflammatory drugs, acetaminophen, and opioids (Amaechi et al., 2021). Morphine, the reference drug for opioid analgesics, produces adequate analgesia due to its high affinity for the μ receptor and is considered the analgesic of choice to treat severe pain (Mathews et al., 2015; Ripplinger et al., 2018). Despite their effectiveness and analgesic potency, high and repeated doses of opioids can result in tolerance, dependence, and withdrawal, in addition to inducing nausea and vomiting, constipation, and respiratory depression (Nafziger and Barkin, 2018; Montgomery, 2022).

Then, in view of the pharmacological potential of 9-aminoacridine derivatives, the present study aimed to evaluate the toxicological, antitumor, antiangiogenic, and immunomodulatory properties of ACS-AZ in the Ehrlich carcinoma model. In addition, the antinociceptive action and role of µ1-opioid receptors in mice were also investigated.

## 2 Material and methods

### 2.1 Drugs and reagents

The following drugs and reagents were acquired in Sigma-Aldrich® (St. Louis, MO, United States): 5-fluorouracil (5-FU), diazepam, naloxonazine, cyclophosphamide, and Tween 80. The anesthetics ketamine and xylazine were obtained from Konig S.A. (Brazil), and MSD Saúde Animal (Brazil), respectively. Other drugs, reagents, and kits comprise: heparin (HEPAMAXS®), ELISA Kit (eBioscience®, Inc. Science Center Drive, San Diego, CA, United States), kits for hematological and biochemical analysis (LABTEST; Lagoa Santa, MG, Brazil), morphine hydrochloride (Merck®), naloxone hydrochloride (Cristália®), formaldehyde 37% (Vetec), dimethylsulfoxide - DMSO (Mallinckrodt Chemicals - Phillipsburg, NJ, United States). The acridine derivative N’-(6-chloro-2-methoxy-acridin-9-yl)-2-cyanoacetohydrazide (ACS-AZ) was synthesized as previously described (Silva et al., 2018).

### 2.2 Animals and tumor cell line

For this study, male and female Swiss albino mice (*Mus musculus*), 28–32 g (about 60 days old) were provided by the Research Institute in Drugs and Medicines of the Federal University of Paraíba (IPeFarM/UFPB), Brazil. They were kept in polyethylene cages (eight animals/cage) at temperatures between 20°C–22°C. The animals had free access to feed and water in a 12 h/12 h off light-dark cycle. For all experiments, after acclimatization, the mice were randomly assigned to either the control or treatment group. Simple randomization method was used. All animals received the same drug solution volume (1 ml/100 g of body weight). Ethical Committee on the Use of Animals from Federal University of Paraíba (CEUA/UFPB) approved all the experimental procedures performed in the present study (CEUA-UFPB 019/2015). Pharmacology and Toxicology Division, CPQBA, UNICAMP (Paulínia, SP, Brazil) provided the tumor cell line we used, Ehrlich carcinoma cell line.

### 2.3 Evaluation of the acute non-clinical toxicity

The Guideline for Testing of Chemicals N. 423 from the Organisation for Economic Co-operation and Development (OECD) was used to assess the acute toxicity. ACS-AZ was administered intraperitoneally (i.p.) to female mice (*n* = 3/group) using different doses in two steps (1st step: 300 mg/kg, and 2nd step: 2000 mg/kg). For each set of experiment, a control group was used (Tween 80 at 12% (v/v) in saline). The lethal dose of 50% (LD_50_) was estimated (OECD, 2002).

#### 2.3.1 Genotoxicity assay

Mammalian erythrocyte micronucleus assay (Test N. 474 from OECD) was used to evaluate the genotoxicity (OECD, 2016). ACS-AZ (150 mg/kg, i.p.) was administered intraperitoneally (i.p.) to female mice, and cyclophosphamide (50 mg/kg, i.p.) was used as a standard drug (*n* = 6/group). Additionally, a control group was used (Tween 80 at 12% (v/v) in saline). To determine the number of micronucleated erythrocytes, peripheral blood samples were collected from the retroorbital plexus after 48 h of treatment. Then, three slides were prepared for each animal by the blood distension technique, and stained with quick panoptic. A total of 2000 erythrocytes *per* slide were counted using an optical microscope (Nikon, Japan) under immersion objective (×100).

#### 2.3.2 Assessment of the antitumor effect on Ehrlich ascites carcinoma model

The mice were randomly distributed into five groups (*n* = 8/group) and inoculated with 4×10^6^ cells/ml of Ehrlich tumor cells (0.5 ml/animal). Twenty-4 hours after tumor inoculation, three groups of mice were treated (i.p.) with ACS-AZ (12.5, 25, or 50 mg/kg/day) for seven consecutive days. The doses and duration of treatments were chosen from literature reports for acridine compounds in the same experimental model (Duarte et al., 2020; Lisboa et al., 2020; Silva et al., 2020). The remaining two groups were treated with 5-Fluorouracil - 5-FU (25 mg/kg), as the standard drug, and vehicle alone (Tween 80 at 12% (v/v) in saline), the control group (Dolai et al., 2012). After 7 days of treatment, blood was collected from the retro-orbital plexus of animals under anesthesia with ketamine (100 mg/kg, i.m.) and xylazine hydrochloride (16 mg/kg, i.p.) to evaluate hematological and biochemical parameters. Then, after euthanasia by cervical dislocation, the volume (ml) of ascitic fluid was collected from the peritoneal cavity, and tumor cells were harvested and counted by Trypan blue assay.

#### 2.3.3 Determination of microvessel density

To assess the antiangiogenic action, animals’ peritoneum from ACS-AZ (50 mg/kg), 5-FU (25 mg/kg), and control groups were cut and photographed. Tumor microvessel density was quantified by the ratio between the area occupied by blood vessels and the total selected area (Agrawal et al., 2011). The AVSOFT® software was used for the analysis.

#### 2.3.4 Quantification of cytokine levels

To quantificate cytokine levels (IL-1β, TNF-α, CCL-2, IL-4, and IL-10), the animals’ ascitic fluid from ACS-AZ (50 mg/kg), 5-FU (25 mg/kg), and control groups were collected. The mouse sandwich ELISA kit for each cytokine contained ELISA plates pre-coated with the monoclonal capture antibody. ELISA kits were used following the manufacturer’s instructions, and standard curves were applied to calculate the cytokine levels (pg/ml).

#### 2.3.5 Quantification of reactive oxygen species

To quantificate ROS levels, we used the 2,7-dichlorodihydrofluorescein diacetate (DCFH-DA) assay (Hasui et al., 1989). Tumor cells were harvested from the animals’ ascitic fluid from ACS-AZ (50 mg/kg), 5-FU (25 mg/kg), and control groups. Then, 2×10^5^ cells were incubated with 200 µl DCFH-DA solution (0,3 mM) in 5% CO_2_ at 37°C for 30 min. Cells were centrifugated (245 *g*, 4°C, 5 min), washed with PBS (Phosphate-Buffered Saline), and the fluorescence of the DCFH-DA-loaded cells was measured by flow cytometry. The excitation filter was 485 nm and the emission filter was 530 nm, and 10,000 events were acquired. The quantification of reactive oxygen species (ROS) was expressed as the average intensity of fluorescence (Silva et al., 2020).

#### 2.3.6 Quantification of nitrite levels

The Griess reaction method was used to calculate the nitrite concentration in the animals’ ascitic fluid from ACS-AZ (50 mg/kg), 5-FU (25 mg/kg), and control groups (Green et al., 1982). A standard curve was applied to calculate the concentrations of sodium nitrite (µM).

#### 2.3.7 Assessment of the toxicity on Ehrlich ascites carcinoma model

Animals’ body weight from ACS-AZ (50 mg/kg), 5-FU (25 mg/kg), and control groups were recorded at the beginning and end of the treatment. In addition, the consumption of water and food was recorded daily for the 7 days of the treatment. The following biochemical and hematological parameters were also evaluated: urea and creatinine levels, and the alanine aminotransferase - ALT and aspartate aminotransferase - AST activities, hemoglobin (Hb) level, red blood cell (RBC) count, hematocrit (Hct), mean corpuscular volume (MCV), mean corpuscular hemoglobin (MCH), mean corpuscular hemoglobin concentration (MCHC), and total and differential leukocyte counts. Livers and kidneys used for histology were fixed in 10% (v/v) formaldehyde, sectioned (3 μm), and stained with hematoxylin–eosin. To evaluate hepatic fibrosis, liver sections were also stained with Gomori’s trichrome.

### 2.4 Assessment of antinociceptive activity

#### 2.4.1 Rota-rod test

A pre-selection of animals was carried out 24 h before the experiment. Only the animals that remained on the device’s rotating bar (Insight^©^, Ribeirão Preto, SP, Brazil) for a period of 60 s (7 rpm) were selected for the test. Then, mice were randomly distributed into three groups (*n* = 8 males/group): ACS-AZ (50 mg/kg, i.p.), diazepam (1 mg/kg, i.p.), and control (Tween 80 at 12% (v/v) in saline). After 30, 60, and 120 min of treatments, the performance of mice was determined by counting the total time that the animals remained on the bar that rotates at 7 rpm up to a total of 3 min. For each animal, up to three falls were allowed (Dunham and Mita, 1953).

#### 2.4.2 Hot plate test

A pre-selection of animals was carried out by placing mice on the hot plate apparatus (Insight^©^, Ribeirão Preto, SP, Brazil) at 55 ± 1°C (Eddy and Leimback, 1953). Only the animals that had a pain response time of less than 10 s were selected. Then, mice were randomly distributed into three groups (*n* = 8 males/group): ACS-AZ (50 mg/kg, i.p.), morphine (6 mg/kg, i.p.), and control (Tween 80 at 12% (v/v) in saline). After 0.5, 1, and 2 h of treatments, mice were individually placed on the hot plate, and the latency time to jump or lick the hind paws was recorded for each animal. To avoid tissue injury, the cut-off time was defined at 30 s (Bastos et al., 2006).

#### 2.4.3 Formalin test

For the formalin test, mice were placed individually in the chamber for 30 min to allow acclimatization. Then, mice were randomly distributed into three groups (*n* = 8 males/group): ACS-AZ (50 mg/kg, i.p.), morphine (6 mg/kg, i.p.), and control (Tween 80 at 12% (v/v) in saline). These treatments were done 0.5 h before the injection of 20 μl of a solution of formalin (2%), subcutaneous at the dorsum of the right hind paw of each animal. Using the formalin test, it is possible to identify an early or first phase of licking activity (0–5 min after formalin injection), and a late or second phase (15–30 min after formalin injection) (Hunskaar et al., 1985).

#### 2.4.4 Opioid system evaluation

For the antagonism study, naloxone, a non-selective opioid receptor antagonist that blocks all receptor subtypes including mu, delta, and kappa was used. Mice were randomly distributed into five groups (*n* = 8 males/group): control (Tween 80 at 12% (v/v) in saline), ACS-AZ (50 mg/kg, i.p.), and morphine (6 mg/kg, i.p.) in the absence or presence of naloxone (5 mg/kg, s.c.) (Jin et al., 2010) at 15 min before the administration of treatments. To evaluate the antinociceptive effect, the formalin test was performed after 30 min of the treatments, as previously described. To assess the involvement of µ1-opioid receptors in the antinociceptive effect it was used naloxonazine, a µ1-opioid receptor antagonist, 15 min before treatments. Five groups of mice (*n* = 8 males/group) were also used: control group (Tween 80 at 12% (v/v) in saline), ACS-AZ (50 mg/kg, i.p.), and morphine (6 mg/kg i.p.) in the absence or presence of naloxonazine (10 mg/kg, s.c.) (Ling et al., 1986; Zarei et al., 2018). After 30 min, the formalin test was performed.

### 2.5 Statistical analysis

Results are expressed as the mean ± standard error of the mean (X±SEM). For all hypothesis tests, the significance level was 0.05 (α = 0.05) and the null hypothesis (H0) was that the independent variable (ACS-AZ or standard drugs) does not interfere with the response, and the alternative hypothesis (H1) was that the independent variable (ACS-AZ or standard drugs) interferes with the measured response. To compare the means of two groups of parametric data the Student’s t-test was performed, whereas to compare the means of three or more groups of parametric data, analysis of variance (One-way ANOVA) followed by Tukey’s post-hoc test was carried out. The differences between the means were considered significant when *p* < 0.05.

For sample size calculation, the expected effect size was obtained from a previous study (Duarte et al., 2020). Using a 1% significance level, Hedge’s of 2.514 (effect size), the statistical power of 90%, and sample loss of 30%, the required sample size was eight mice per group.

## 3 Results

### 3.1 Toxicological assays

#### 3.1.1 Acute non-clinical toxicity

No deaths were recorded during the initial administration of 300 mg/kg of ACS-AZ. A new experiment with the same dose must be done according to the guideline N. 423 from OECD. Again, no death was recorded. Thus, we applied the dose of 2000 mg/kg and all the animals died. Then, ACS-AZ can be classified into category 4 (LD_50_ cut-off value 500 mg/kg, i.p.), according to the Globally Harmonized System (GHS) for the classification of chemicals.

#### 3.1.2 Genotoxicity

No changes in the number of micronucleated erythrocytes from mice’s peripheral blood were detected after the ACS-AZ (150 mg/kg) treatment (4.50 ± 0.56) compared to the control (6.00 ± 0.57). On the other hand, treatment with cyclophosphamide (50 mg/kg) increased the number of micronucleated erythrocytes (18.40 ± 0.52; *F*_2,18_ = 11.52, *p* < 0.05) in relation to control (Table 1).

**TABLE 1:** Micronucleus test in peripheral blood of mice treated with a single dose of ACS-AZ or cyclophosphamide.

Groups	Dose (mg/kg)	Number of micronucleated erythrocytes
Control	**–**	6.00 ± 0.57
Cyclophosphamide	50	18.40 ± 0.52^a^
ACS-AZ	150	4.50 ± 0.56

The table shows the mean ± SEM (standard error of the mean) for the number of micronucleated erythrocytes in peripheral blood of mice. (*n* = 6, ^a^ *p* < 0.05 vs. control analyzed by one-way ANOVA followed by Tukey test). ACS-AZ: N'-(6-chloro-2-methoxyacridin-9-yl)-2-cyanoacetohydrazide.

### 3.2 Antitumor activity on Ehrlich ascites carcinoma model

**FIGURE 2:**
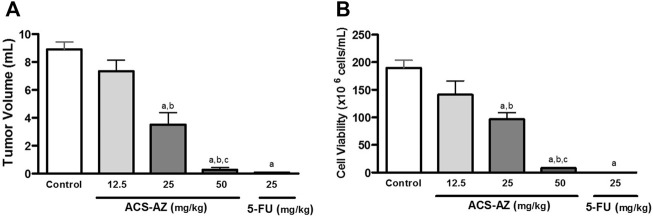
Effects of a 7-day treatment with ACS-AZ (12.5, 25, and 50 mg/kg) or 5-FU (25 mg/kg) by intraperitoneal route on **(A)** tumor volume (ml), and **(B)** cell viability (x10^6^ cells/mL) in Ehrlich ascites carcinoma-bearing mice. Cell viability was determined by Trypan blue assay. The results represent the mean ± SEM (*n* = 8) analyzed by one-way ANOVA followed by Tukey test: ^a^ *p* < 0.05 vs. control group; ^b^ *p* < 0.05 vs. 12.5 mg/kg ACS-AZ group; ^c^ *p* < 0.05 vs. 25 mg/kg ACS-AZ group. ACS-AZ: N'-(6-chloro-2-methoxyacridin-9-yl)-2-cyanoacetohydrazide; 5-FU: 5-fluorouracil.

ACS-AZ was significantly effective in reducing tumor volume and cell viability. At doses of 25 and 50 mg/kg, ACS-AZ induced a decrease in tumor volume (3.50 ± 0.88 ml and 0.30 ± 0.15 ml, respectively; *F*_4,25_ = 49.42, *p* < 0.05 for both) compared to control (8.90 ± 0.50 ml). Furthermore, we observed a significant reduction in tumor cell viability for 96.70 ± 11.80 × 10^6^ cells/mL and 8.00 ± 0.10 × 10^6^ cells/mL (*F*_4,27_ = 37.48, *p* < 0.05 for both) at 25 and 50 mg/kg ACS-AZ, respectively, in comparison to control (189.90 ± 13.70 × 10^6^ cells/mL). The standard drug 5-FU (25 mg/kg) also reduced both parameters (*p* < 0.05). The data are shown in Figure 2.

#### 3.2.1 Antiangiogenic effect—microvessel density

**FIGURE 3:**
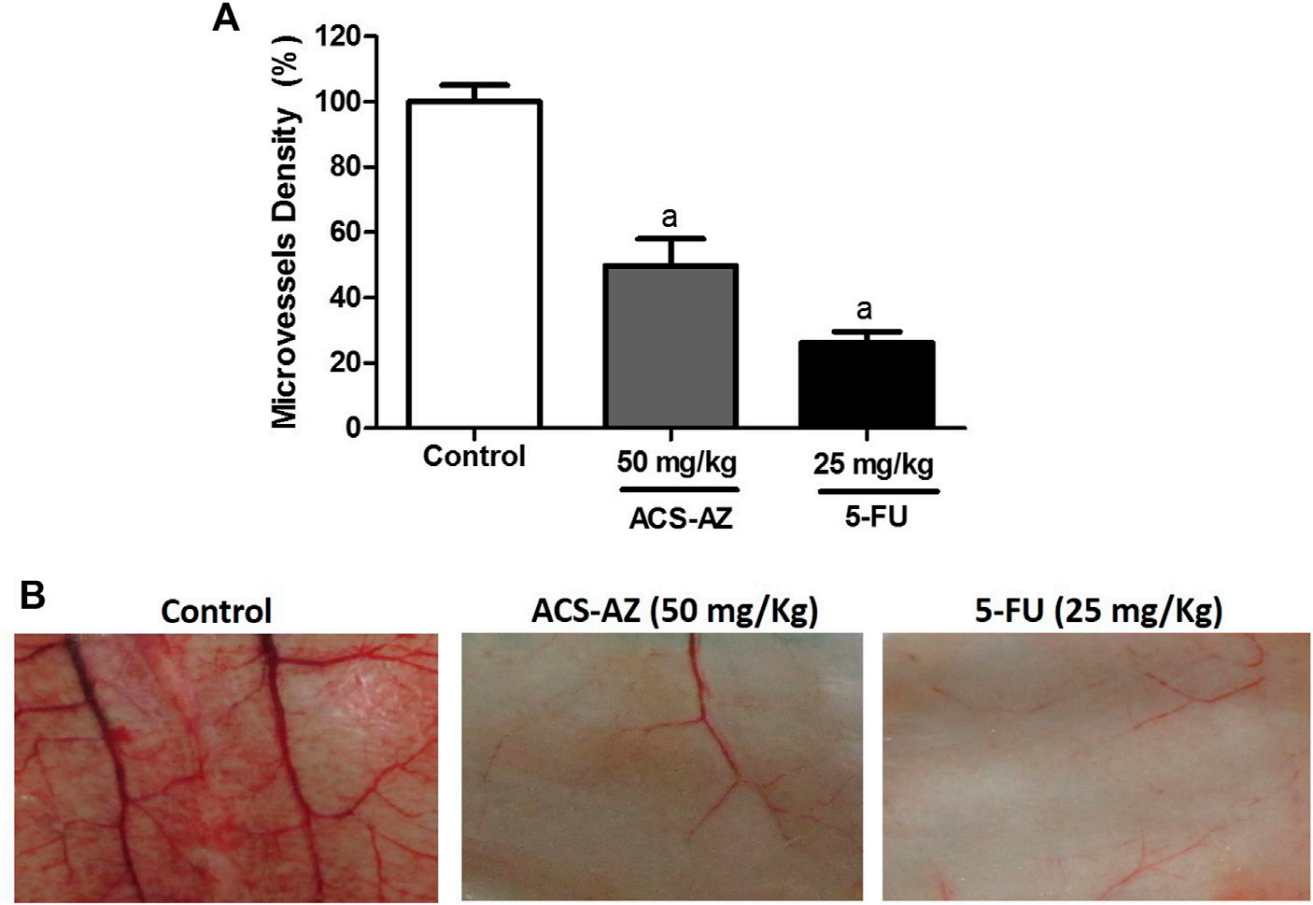
Effect of a 7-day treatment with ACS-AZ (50 mg/kg) or 5-FU (25 mg/kg) by intraperitoneal route on microvessel density (%) in Ehrlich ascites carcinoma-bearing mice. Animals’ peritoneum were cut and photographed. **(A)** Microvessel density (%) represents the blood vessel area per field in selected vascularized areas divided by the whole area. **(B)** Representative images of cut from animal’s peritoneum into a standard size (1 cm × 1 cm). The results represent the mean ± SEM (*n* = 8) analyzed by one-way ANOVA followed by Tukey test: ^a^ *p* < 0.05 vs. control. ACS-AZ: N'-(6-chloro-2-methoxyacridin-9-yl)-2-cyanoacetohydrazide; 5-FU: 5-fluorouracil.

After 7 days of treatment, ACS-AZ decreased peritoneal angiogenesis in Ehrlich tumor-bearing mice (Figure 3). At the dose of 50 mg/kg, ACS-AZ significantly reduced the microvessel density (49.78 ± 8.00%; *F*_2,15_ = 49.97, *p* < 0.05) and 5-FU (26.30 ± 3.38%; *p* < 0.05) compared to the control (100 ± 5.09%).

#### 3.2.2 Quantification of cytokine levels

**FIGURE 4:**
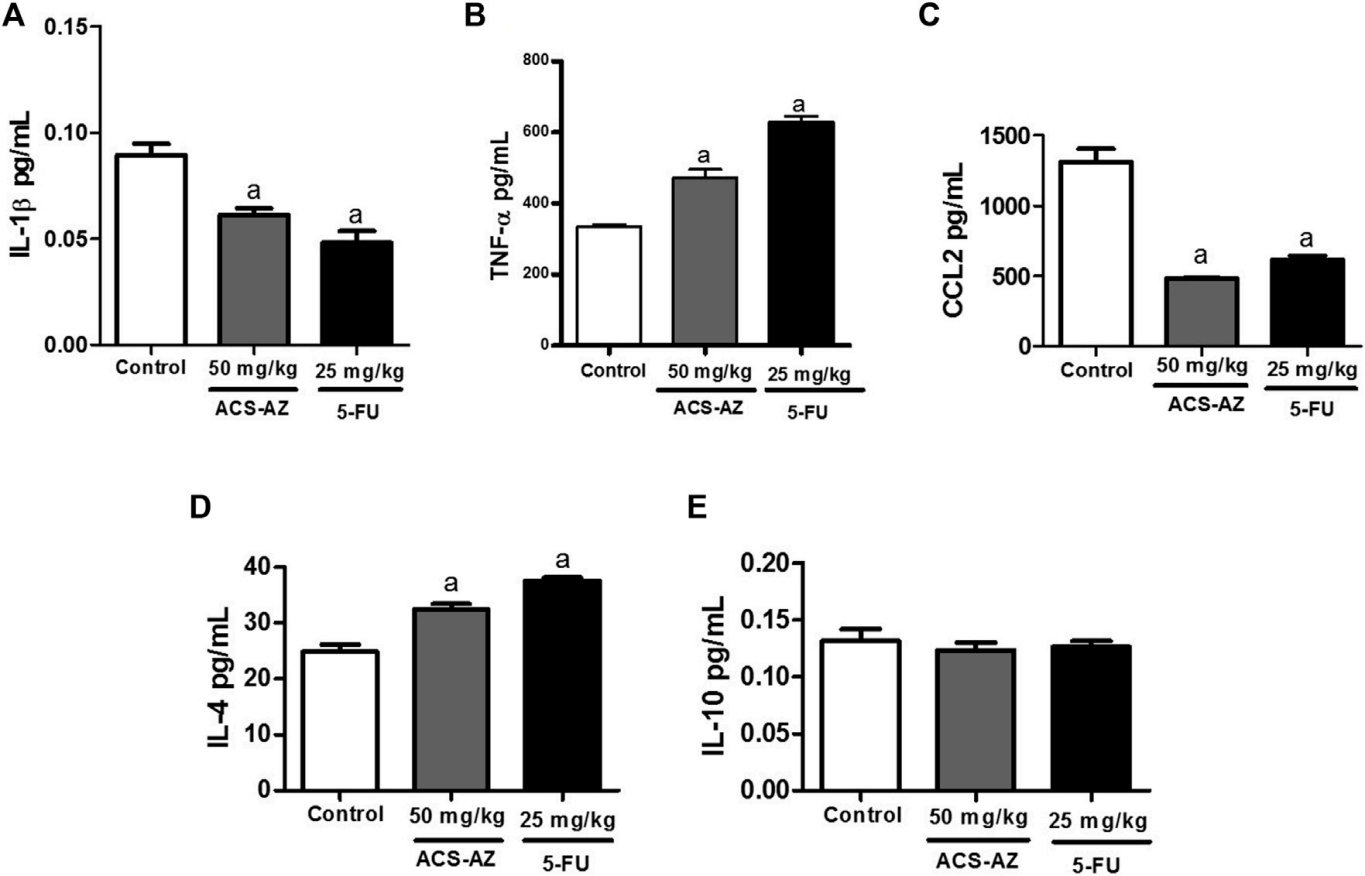
Effect of a 7-day treatment with ACS-AZ (50 mg/kg) or 5-FU (25 mg/kg) by intraperitoneal route on cytokine levels from the ascitic fluid of Ehrlich ascites carcinoma-bearing mice. Levels (pg/ml) of IL-1β **(A)**, TNF-α **(B)**, CCL2 **(C)**, IL-4 **(D)**, and IL-10 **(E)** were determined by mouse sandwich ELISA kits for each cytokine containing ELISA plates pre-coated with the monoclonal capture antibody. The results represent the mean ± SEM (*n* = 8) analyzed by one-way ANOVA followed by Tukey test: ^a^ *p* < 0.05 vs. control. ACS-AZ: N'-(6-chloro-2-methoxyacridin-9-yl)-2-cyanoacetohydrazide; 5-FU: 5-fluorouracil; IL: interleukin; TNF-α: tumor necrosis factor-α; CCL-2: CC motif chemokine ligand 2.

ACS-AZ (50 mg/kg) reduced (*p* < 0.05) the IL-1β levels (0.06 ± 0.01 pg/ml; *F*_2,27_ = 20.23) and CCL2 chemokine (485.40 ± 2.52 pg/ml; *F*_2,28_ = 49.98) compared to the control (0.09 ± 0.01 pg/ml and 1,313.0 ± 90.73 pg/ml, respectively). Regarding TNF-α levels, a significant increase (472.80 ± 22.90 pg/ml; *F*_2,26_ = 75.63, *p* < 0.05) was observed compared to the control group (334.50 ± 5.90 pg/ml). For anti-inflammatory cytokines, there was an increase in IL-4 levels (32.40 ± 1.0 pg/ml; *F*_2,23_ = 39.45, *p* < 0.05) in animals treated with ACS-AZ in relation to control (24.82 ± 1.27 pg/ml). Finally, there was no significant change in IL-10 levels. Similar results were recorded for 5-FU group. The data are shown in Figure 4.

#### 3.2.3 ROS and nitrite quantification

**FIGURE 5:**
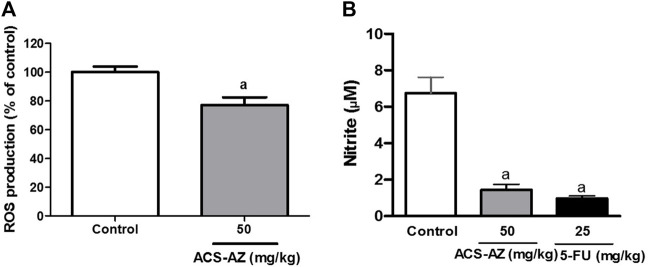
Effect of a 7-day treatment with ACS-AZ (50 mg/kg) or 5-FU (25 mg/kg) by intraperitoneal route on Reactive Oxigen Species—ROS (% of control) **(A)** and nitrite (µM) **(B)** quantification from the ascitic fluid of Ehrlich ascites carcinoma-bearing mice. The results represent the mean ± SEM (*n* = 8) analyzed by Student’s t-test for DCFH-DA assay, and one-way ANOVA followed by Tukey test for Griess assay: ^a^ *p* < 0.05 vs. control. ACS-AZ: N'-(6-chloro-2-methoxyacridin-9-yl)-2-cyanoacetohydrazide; 5-FU: 5-fluorouracil.

ACS-AZ (50 mg/kg) reduced the reactive oxygen species (ROS) production (77.11 ± 5.41%; *p* < 0.05), compared to the control group (100.0 ± 3.9%) in the DCFH-DA assay (Figure 5A). Additionally, treatment with ACS-AZ (50 mg/kg) also reduced the nitrite concentration (1.44 ± 0.31 µM; *F*_2,30_ = 13.08, *p* < 0.05), measured using Griess reagent, and 5-FU drug (0.97 ± 0.13 µM; *p* < 0.05) compared to the control group (6.74 ± 0.88 µM) (Figure 5B).

#### 3.2.4 Toxicity in Ehrlich tumor transplanted mice

Water consumption was significantly reduced after 50 mg/kg ACS-AZ treatment (40.83 ± 1.54 ml; *F*_2,16_ = 17.11, *p* < 0.05) compared to the control (56.67 ± 2.79 ml); however, no changes in feed consumption and animal body weights were observed. Similar results were observed for 5-FU (Table 2). All biochemical and hematological parameters were also preserved after ACS-AZ treatment (Table 3). Inversely, the standard drug 5-FU reduced (*p* < 0.05) several hematological markers of the red blood series, such as erythrocyte count (5.84 ± 0.16 10^6^/mm^3^; *F*_2,22_ = 115.0), hemoglobin (12.31 ± 0.28 g/dl; *F*_2,21_ = 20.04), and hematocrit (35.0 ± 1.10%; *F*_2,21_ = 68.19), and induced a significant increase (*p* < 0.05) in MCH (22.0 ± 0.43 pg; *F*_2,21_ = 232.2) and MCHC (36.0 ± 0.97 g/dl; *F*_2,21_ = 232.2) compared to the control (9.61 ± 0.14 10^6^/mm^3^; 14.83 ± 0.32 g/dl; 50.76 ± 1.17%; 14.73 ± 0.15 pg; and 29.10 ± 0.18 g/dl, respectively). Concerning the hematological markers of the white blood series, 5-FU reduced (*p* < 0.05) the total leukocyte count (3.66 ± 0.29 10^3^/mm^3^) and the percentage of segmented (9.40 ± 0.90%; *F*_2,16_ = 32.76) and monocytes (1.0 ± 0.30%; *F*_2,15_ = 9.527), in addition to increasing (*p* < 0.05) the lymphocyte percentage (88.0 ± 1.10%; *F*_2,16_ = 32.53) compared to the control (13.13 ± 0.95 10^3^/mm^3^, 19.0 ± 1.10%, 2.90 ± 0.30% and 77.0 ± 1.0%, respectively) (Table 3).

**TABLE 2:** Effects of a 7-day treatment with ACS-AZ or 5-FU on body weight and feed and water consumption of Ehrlich ascites carcinoma-bearing mice.

Groups	Water intake (ml)	Food intake (g)	Starting weight (g)	Final weight (g)
Control	56.67 ± 2.79	27.83 ± 3.81	32.20 ± 0.31	28.08 ± 0.85
ACS-AZ (50 mg/kg)	40.83 ± 1.54^a^	21.17 ± 2.0	30.88 ± 0.55	27.0 ± 1.10
5-FU (25 mg/kg)	37.86 ± 2.64^a^	19.71 ± 0.84	30.67 ± 0.67	28.67 ± 0.33

The table shows the mean ± SEM (standard error of the mean) for body weight and feed and water consumption of mice. (*n* = 8, ^a^ *p* < 0.05 vs. control analyzed by one-way ANOVA followed by Tukey test). ACS-AZ: N'-(6-chloro-2-methoxyacridin-9-yl)-2-cyanoacetohydrazide; 5-FU: 5-fluorouracil.

**TABLE 3:** Biochemical and hematological parameters of peripheral blood of Ehrlich ascites carcinoma-bearing mice after a 7-day treatment with ACS-AZ or 5-FU.

Parameters	Control	ACS-AZ (50 mg/kg)	5-FU (25 mg/kg)
Biochemical parameters			
ALT (U/L)	165.90 ± 12.61	160. 0 ± 8.50	154.60 ± 8.0
AST (U/L)	170.10 ± 10.77	174.30 ± 4.40	176.60 ± 3.34
Urea (mg/dl)	35.38 ± 3.0	35.25 ± 2.50	35.38 ± 3.10
Creatinine (mg/dl)	0.84 ± 0.0	0.98 ± 0.10	0.81 ± 0.0
Hematological parameters			
Erythrocytes (10^6^/mm^3^)	9.61 ± 0.14	10.08 ± 0.31^b^	5.84 ± 0.16^a^
Hemoglobin (g/dl)	14.83 ± 0.32	14.45 ± 0.30^b^	12.31 ± 0.28^a^
Hematocrit (%)	50.76 ± 1.17	50.90 ± 1.0^b^	35.0 ± 1.10^a^
MCV (fm^3^)	56.61 ± 3.65	52.23 ± 1.46^b^	63.80 ± 1.68
MCH (pg)	14.73 ± 0.15	14.24 ± 0.18^b^	22.0 ± 0.43^a^
MCHC (g/dl)	29.10 ± 0.18	28.21 ± 0.30^b^	36.0 ± 0.97^a^
Total Leukocytes (10^3^/mm^3^)	13.13 ± 0.95	15.65 ± 0.95^b^	3.66 ± 0.29^a^
Lymphocytes (%)	77.0 ± 1.0	73.70 ± 1.40^b^	88.0 ± 1.10^a^
Neutrophils (%)	19.0 ± 1.10	22.80 ± 1.10^b^	9.40 ± 0.90^a^
Monocytes (%)	2.90 ± 0.30	2.80 ± 0.40^b^	1.0 ± 0.30^a^
Eosinophils (%)	1.0 ± 0.30	0.60 ± 0.20	1.30 ± 0.20

The table shows the mean ± SEM (standard error of the mean) for biochemical and hematological parameters of mice peripheral blood. (*n* = 8, ^a^ *p* < 0.05 vs. control; ^b^ *p* < 0.05 vs. 5-FU group analyzed by one-way ANOVA followed by Tukey test). ACS-AZ: N'-(6-chloro-2-methoxyacridin-9-yl)-2-cyanoacetohydrazide; 5-FU: 5-fluorouracil.

**FIGURE 6:**
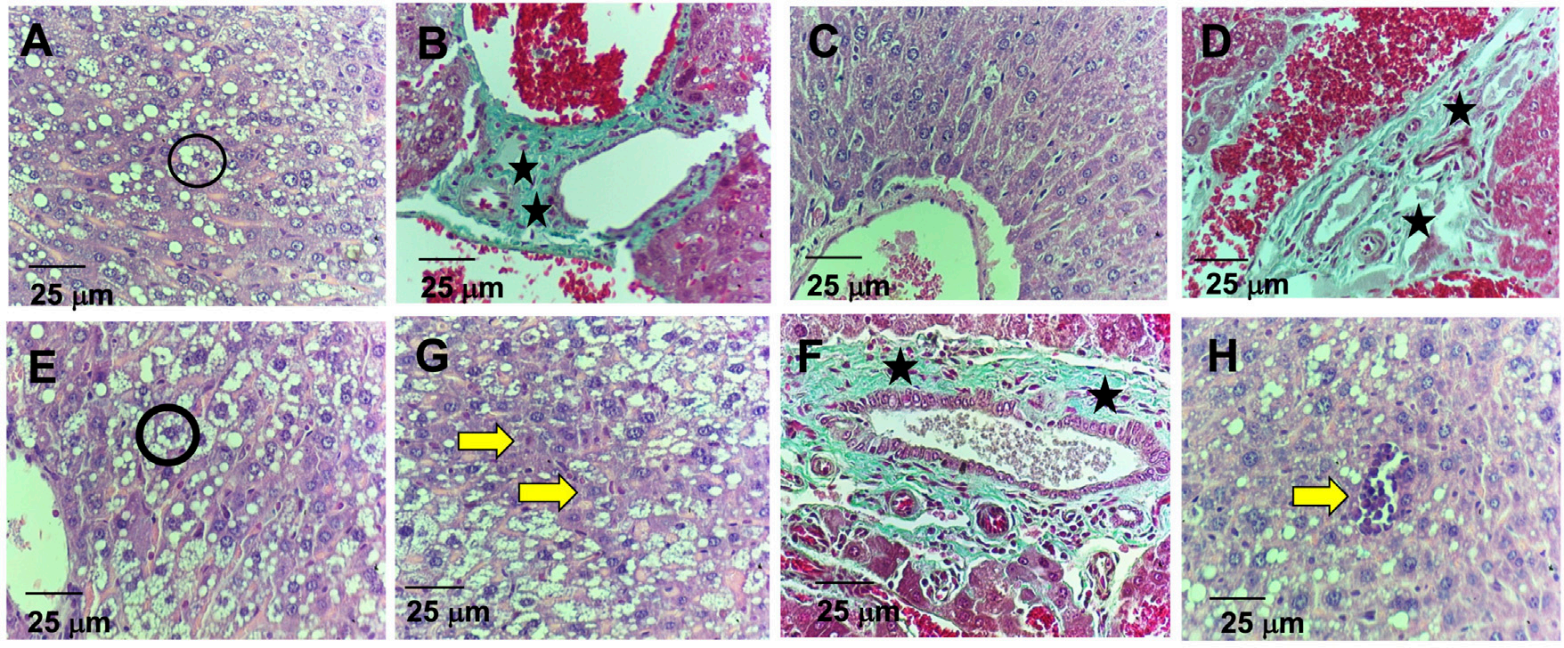
Histological study on liver of Ehrlich ascites carcinoma-bearing mice exposed to ACS-AZ (50 mg/kg) or 5-FU (25 mg/kg) by intraperitoneal route for 7 days. **(A,B)** Control. **(A)** Hepatocyte vacuolation (black circle). **(B)** Connective tissue areas in the portal region (black star). **(C,D)** Group 5-FU. **(C)** Preserved cords of hepatocytes. **(D)** Connective tissue areas in the portal region (black star). **(E,F,G,H)** Group ACS-AZ (50 mg/kg). **(E)** Hepatocyte vacuolation (black circle). **(F)** Connective tissue areas in the portal region (black asterisk) **(G)** Hepatic necrosis (yellow arrow). **(H)** mild inflammatory infiltrates (yellow arrow). **(A,C,E,G,H)**- HE 400x; **(B,D,F)**–Gomori’s trichrome. 400×. ACS-AZ: N'-(6-chloro-2-methoxyacridin-9-yl)-2-cyanoacetohydrazide; 5-FU: 5-fluorouracil.

**FIGURE 7:**
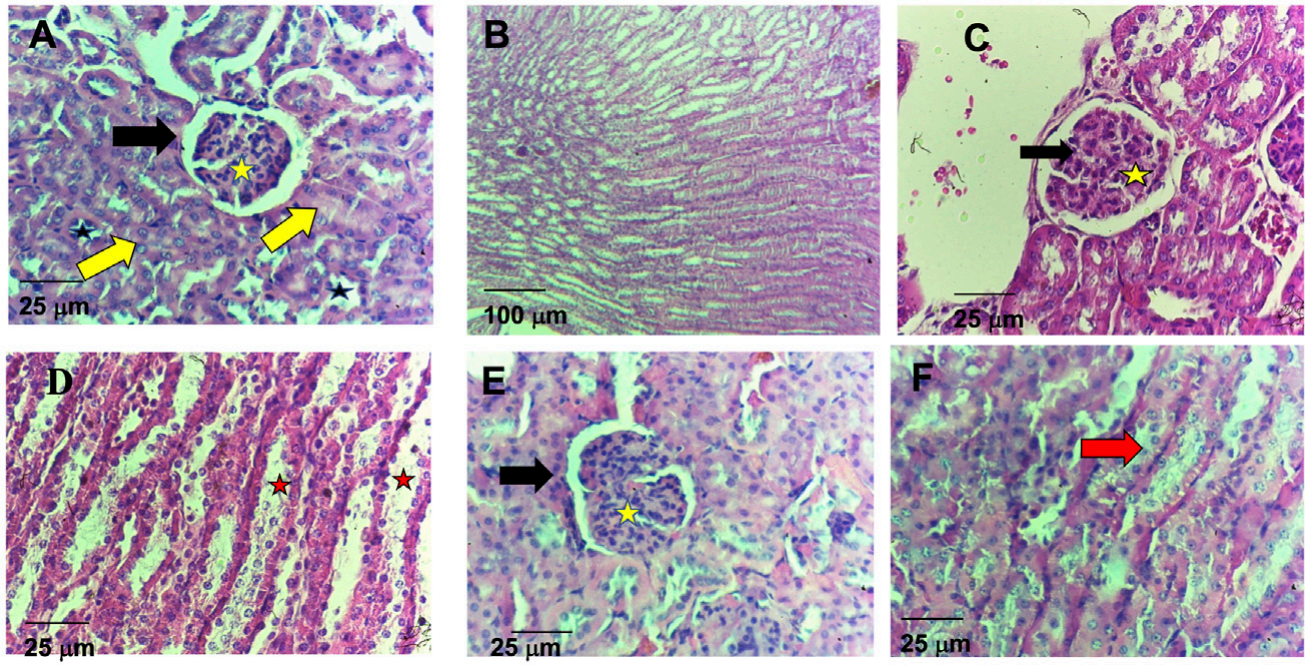
Histological study on kidney of Ehrlich ascites carcinoma-bearing mice exposed to ACS-AZ (50 mg/kg) or 5-FU (25 mg/kg) by intraperitoneal route for 7 days. **(A,B)** Control. **(A)** Preserved renal cortex with glomerulus surrounded by Bowman’s capsule (black arrow) with internal capillary tuft (yellow star), proximal (yellow arrow), and distal (black star) tubules. **(B)** Preserved collecting ducts in the renal medulla. **(C,D)** Group 5-FU. **(C)** In the renal cortex, conserved glomeruli (black arrow) and tubules (yellow star). **(D)** Visible light in the medullary region (red star). **(E,F)** Group ACS-AZ (50 mg/kg). **(E)** Conserved renal cortex with glomerulus surrounded by Bowman’s capsule (black arrow), internal capillary tuff (yellow star), and proximal and distal tubules. **(F)** Conserved tubular epithelium (red arrow). **(A,C,D,E,F)** HE, 400×; **(B)**HE, 100x. ACS-AZ: N'-(6-chloro-2-methoxyacridin-9-yl)-2-cyanoacetohydrazide; 5-FU: 5-fluorouracil.

Regarding the histological analysis of animals’ livers from the control group, it was detected hepatocyte vacuolation in the liver parenchyma (Figure 6A), suggesting hepatic steatosis. Additionally, increased amounts of connective tissue in the portal area were detected by the Gomori’s trichrome staining (Figure 6B), which is associated with perivascular fibrosis. For the 5-FU group, the animals’ livers presented normal architecture and morphology of the hepatocytes and sinusoids and the central vein (Figure 6C). However, increased amounts of connective tissue in the portal area (perivascular fibrosis) were also recorded (Figure 6D). In the livers of the animals treated with ACS-AZ (50 mg/kg), it was observed areas of isolated acidophilic hepatocytes, suggesting coagulative necrosis (Figure 6G), mild inflammatory infiltrate made up of mononuclear cells (Figure 6H), similar hepatocyte vacuolation in the liver parenchyma (hepatic steatosis) (Figure 6E), and connective tissue deposition in the portal area (perivascular fibrosis) (Figure 6F). Regarding the histological analysis of the animals’ kidneys, it was observed that all of the treatments (control, ACS-AZ, and 5-FU) did not change the histological architecture of the tissue (Figures 7A–F). For all the groups, the kidney tissue structures, such as the renal cortex with glomerulus surrounded by Bowman’s capsule, and proximal and distal tubules, were preserved (Figures 7A,C,D). Similarly, collecting ducts were preserved in the renal medulla, as observed by the visible light (Figures 7B,C,D).

### 3.3 Antinociceptive effect

#### 3.3.1 Motor coordination test

ACS-AZ treatment (50 mg/kg) had no effect on the rotating bar permanence times at 30 min (175.60 ± 2.0 s; *F*_2,13_ = 5.283); 60 min (178.0 ± 0.7 s; *F*_2,21_ = 11.36), and 120 min (175.50 ± 1.58 s; *F*_2,20_ = 1.566) after treatment as compared to the control (175.80 ± 2.32 s; 176.50 ± 1.47 s and 177.40 ± 0.80 s, respectively) (Table 4). As expected, diazepam (1 mg/kg) significantly reduced (*p* < 0.05) the rotating bar permanence times observed at 30 min (56.67 ± 0.92 s) and 60 min (98.88 ± 0.58 s) after treatment (Table 4).

**TABLE 4:** Rota-rod test after 30, 60, and 120 min of ACS-AZ (50 mg/kg) and Diazepam (1 mg/kg) treatments.

Groups	Dose (mg/kg)	Time of permanence in the rotating bar (s)		
		30 min	60 min	120 min
Control	-	175.80 ± 2.32	176.50 ± 1.47	177.40 ± 0.80
ACS-AZ	50	175.60 ± 2.00^b^	178.0 ± 0.70^b^	175.50 ± 1.58
Diazepam	1	56.67 ± 0.92^a^	98.88 ± 0.58^a^	172.60 ± 2.00

The table shows the mean ± SEM (standard error of the mean) for time of permanence in the rotating bar (s). (*n* = 8, ^a^ *p* < 0.05 vs. control; ^b^ *p* < 0.05 vs. diazepam group analyzed by one-way ANOVA followed by Tukey test). ACS-AZ: N'-(6-chloro-2-methoxyacridin-9-yl)-2-cyanoacetohydrazide.

#### 3.3.2 Formalin-induced nociception

**FIGURE 8:**
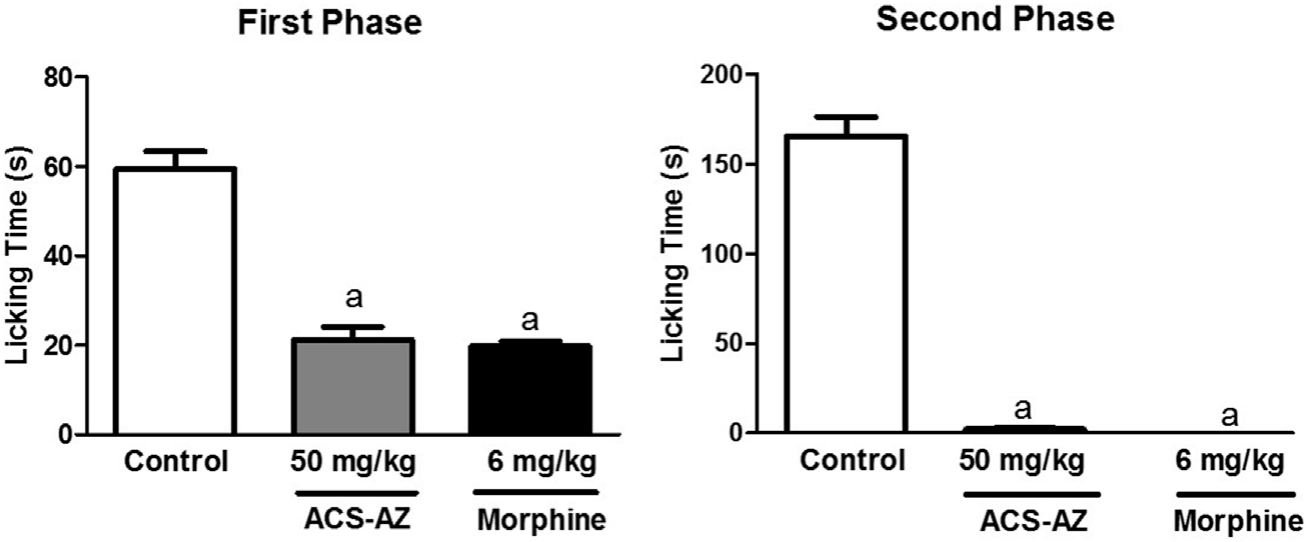
Effect of ACS-AZ (50 mg/kg) or morphine (6 mg/kg) pretreatment by intraperitoneal route on the first and second phases of the formalin-induced nociception test. The lick time (seconds) after administration of formalin solution into the mice dorsal surface of the right hind paw was recorded for each animal. The results represent the mean ± SEM (*n* = 8) analyzed by one-way ANOVA followed by Tukey test: ^a^ *p* < 0.05 vs. control. ACS-AZ: N'-(6-chloro-2-methoxyacridin-9-yl)-2-cyanoacetohydrazide.

ACS-AZ (50 mg/kg) reduced (*p* < 0.05) the paw licking time in the first phase, neurogenic pain (21.22 ± 2.89 s; *F*_2,24_ = 62.63), and morphine (6 mg/kg) (19.78 ± 1.0 s), compared to the control group (59.56 ± 3.86 s). During the second phase of the test (inflammatory pain), ACS-AZ also reduced paw licking time significantly (2.0 ± 0.08 s; *F*_2,26_ = 279.6, *p* < 0.05) compared to the control (165.70 ± 10.41 s). As expected, morphine decreased the same parameter (0.06 ± 0.01 s; *p* < 0.05) (Figure 8).

#### 3.3.3 Hot plate test

**FIGURE 9:**
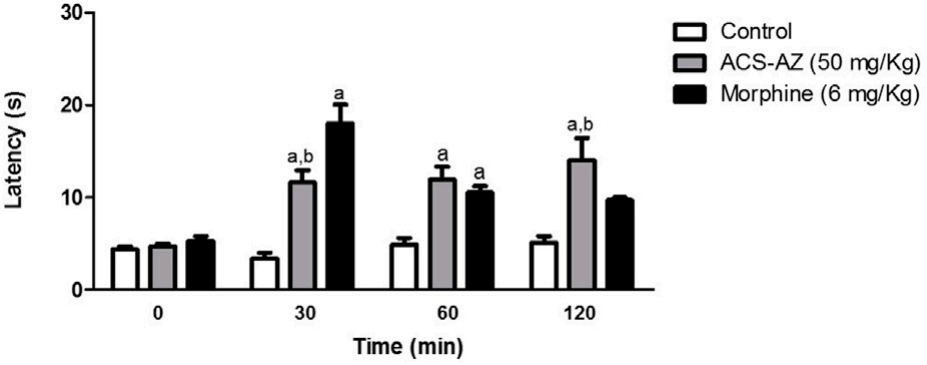
Effect of ACS-AZ (50 mg/kg) or morphine (6 mg/kg) by intraperitoneal route in the hot plate test performed at 30, 60, and 120 min after treatment. Latency time (seconds) to jump or lick the hind paws was recorded for each animal. The results represent the mean ± SEM (*n* = 8) analyzed by one-way ANOVA followed by Tukey test: ^a^ *p* < 0.05 vs. control; ^b^ *p* < 0.05 vs. morphine group. ACS-AZ: N'-(6-chloro-2-methoxyacridin-9-yl)-2-cyanoacetohydrazide.

As assessed by the hot plate test, ACS-AZ (50 mg/kg) significantly (*p* < 0.05) induced an increase in the latency time for nociceptive response to the thermal stimulus. This effect started at 30 min and spanned throughout the observation period of 120 min. The latency time increased from 3.40 ± 0.62 s, 4.90 ± 0.72 s, and 5.12 ± 0.71 s (control values) to 11.60 ± 1.31 s (*F*_2,21_ = 13.30), 11.90 ± 1.40 s (*F*_2,21_ = 14.36), and 13.96 ± 2.44 s (*F*_2,21_ = 12.36) (ACS-AZ values) at 30, 60, and 90 min after treatment, respectively. Morphine exerted a similar effect, although the effect of the acridine compound lasted longer (Figure 9).

#### 3.3.4 Involvement of the opioid system in the antinociceptive effect

**FIGURE 10:**
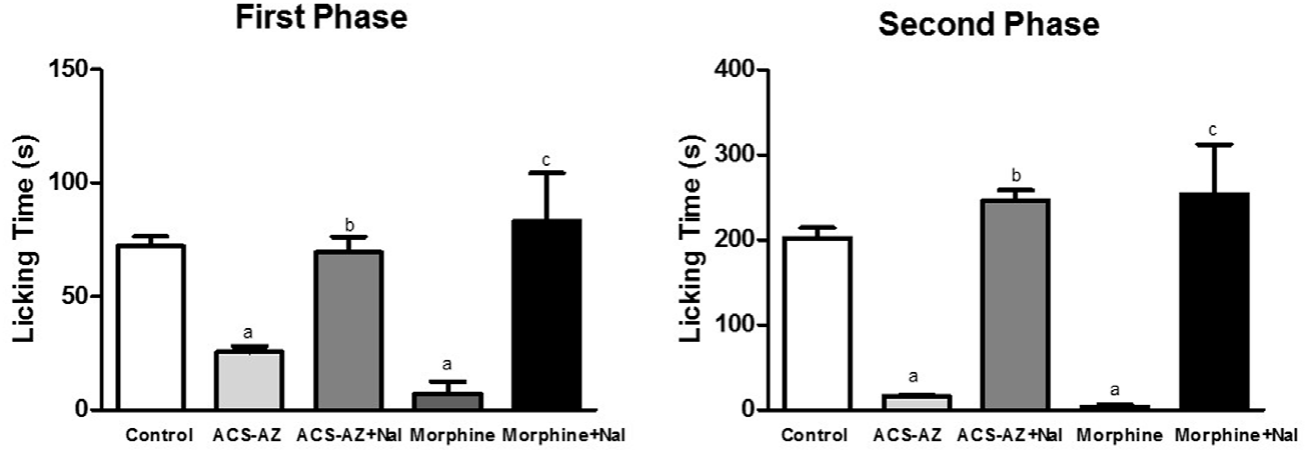
Effect of pretreatment with naloxone (5 mg/kg) on antinociception induced by ACS-AZ (50 mg/kg) or morphine (6 mg/kg) at the first and second phases of the formalin-induced nociception. The lick time (seconds) after administration of formalin solution into the mice dorsal surface of the right hind paw was recorded for each animal. The results represent the mean ± SEM (*n* = 8) analyzed by one-way ANOVA followed by Tukey test: ^a^ *p* < 0.05 vs. control; ^b^ *p* < 0.05 vs. group that received only ACS-AZ; ^c^ *p* < 0.05 vs. group that received only morphine. ACS-AZ: N'-(6-chloro-2-methoxyacridin-9-yl)-2-cyanoacetohydrazide; Nal: naloxone.

The pretreatment with naloxone (opioid antagonist) significantly increased (*p* < 0.05) the paw licking time and reversed the antinociceptive activity of the ACS-AZ (50 mg/kg) in the first (69,5 ± 6,9 s; *F*_4,34_ = 6.038) and second (246,6 ± 11,9 s; *F*_4,36_ = 16.51) phases of the formalin test compared to the groups that received only ACS-AZ (1st phase: 25.5 ± 2.6 s; 2nd phase: 16.5 ± 1.1 s). As expected, naloxone also reversed the antinociceptive effect induced by morphine (6 mg/kg) (1st phase: 83,0 ± 8,1 s and 2nd phase: 246,6 ± 22,4 s) compared to the only morphine groups (Figure 10).

#### 3.3.5 Role of µ1-opioid receptors

**FIGURE 11:**
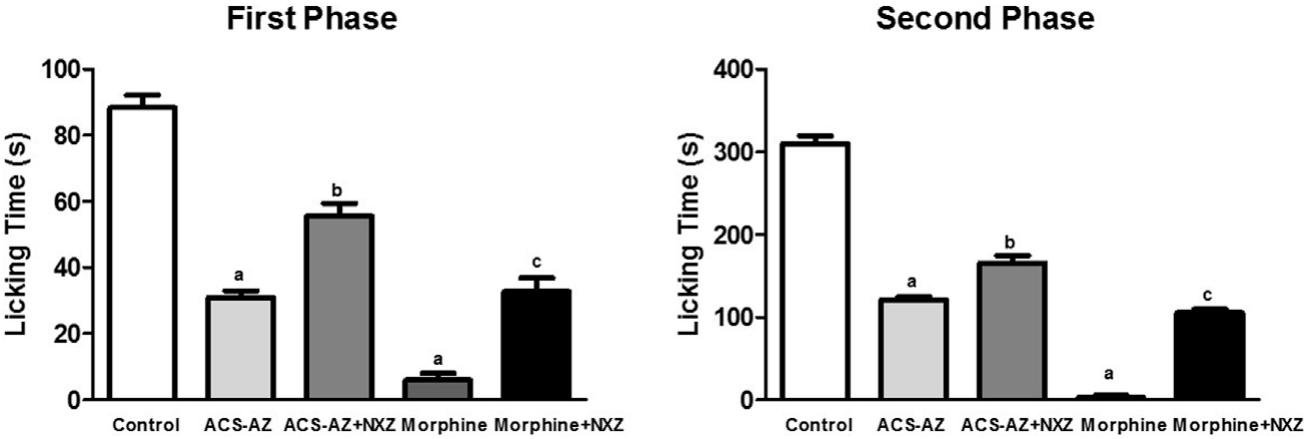
Effect of pretreatment with naloxonazine (10 mg/kg) on antinociception of ACS-AZ (50 mg/kg) and morphine (6 mg/kg), in the first and second phases of formalin-induced nociception. The lick time (seconds) after administration of formalin solution into the mice dorsal surface of the right hind paw was recorded for each animal. The results represent the mean ± SEM (*n* = 8) analyzed by one-way ANOVA followed by Tukey test: ^a^ *p* < 0.05 vs. control; ^b^ *p* <0.05 compared to the group that received only ACS-AZ; ^c^ *p* <0.05 compared to the group that received only morphine. ACS-AZ: N'-(6-chloro-2-methoxyacridin-9-yl)-2-cyanoacetohydrazide; NXZ: naloxonazine.

The pretreatment with naloxonazine (10 mg/kg), a potent irreversible μ1-opioid receptor antagonist, induced a significant increase (*p* < 0.05) in the paw licking time of the animals treated with ACS-AZ (50 mg/kg). We observed a partial block of the antinociceptive effect induced by ACS-AZ at the first (55.63 ± 3.85 s; *F*_4,35_ = 11.76) and second (165.80 ± 8.66 s; *F*_4,40_ = 338.4) phases compared to the group that received only ACS-AZ (1st phase: 31.0 ± 2.0 s; 2nd phase: 121.60 ± 3.35 s). The antinociceptive effect of morphine (6 mg/kg) was also partially reversed by naloxonazine (1st phase: 32.75 ± 1.45 s and 2nd phase: 105.80 ± 1.42 s) compared to the group that received only morphine (Figure 11).

## 4 Discussion

The acridine scaffold has attracted attention in oncology since its DNA affinity and intercalative properties make it an important pharmacophore for the design of antitumor drugs targeting DNA (Denny, 2002; Belmont and Dorange, 2008; Fu et al., 2017; Lambert et al., 2018; Duarte et al., 2020; Oyedele et al., 2020; Majhi, 2022; Silva et al., 2019). In this regard, we can highlight the 9-aminoacridine derivatives, which have shown potential anticancer, antinociceptive, and against neurodegenerative diseases and protozoal parasites. In continuation of our efforts in search of potential acridine derivatives, we tested the N’-(6-chloro-2-methoxyacridin-9-yl)-2-cyanoacetohydrazide (ACS-AZ), a 9-aminoacridine derivative with antimalarial effect. Herein, we report the toxicological, antitumor, and antinociceptive effects of ACS-AZ.

An acute non-clinical toxicity test was performed to establish safe doses for *in vivo* pharmacological assays (OECD, 2002). ACS-AZ presented low toxicity under the experimental conditions tested, with LD_50_ estimated at around 500 mg/kg (i.p.). In general, acridine derivatives are molecules with different toxicity profiles, and both low (Mangueira et al., 2017) and high (Pan et al., 2007) acute toxicity have been reported. Literature reports have shown that if the minimum effective dose of the drug is three times lower than the LD_50_, the drug can be considered a suitable candidate for additional studies. Thus, we chose ACS-AZ safe doses for the antinociceptive and antitumor activity assays in mice (Auletta, 2002).

Furthermore, we evaluated the *in vivo* genotoxic potential of ACS-AZ (150 mg/kg) using the mice’s peripheral blood by the micronucleus assay. Genotoxicity is generally evaluated for testing the safety of new drugs, and the micronucleus test in rodents is one of the best and most popular. In this cytogenetic assay, erythrocytes from the peripheral blood or bone marrow are used to identify chemical damage to the chromosomes or mitotic apparatus of rodent cells. The potential genotoxicity of a drug is suggested when an increase in the frequency of micronucleated erythrocytes in treated animals is detected (Sommer et al., 2020). Herein, ACS-AZ did not change the number of micronucleated erythrocytes in peripheral blood, which allows us to infer that ACS-AZ does not induce a genotoxic effect. These results corroborate literature data, which showed that other acridine derivatives, such as amsacrine and ACS-AZ10, have no genotoxic effects in the micronucleus assay (Lynch et al., 2008; Mangueira et al., 2017). The absence of mutagenic activity is a crucial property in non-clinical studies of new and/or potential anticancer agents (Recio et al., 2010; Mello-Andrade et al., 2018).

Once the safety of ACS-AZ was determined, we evaluated its antitumor potential. The Ehrlich ascites carcinoma (EAC) model, a spontaneous murine mammary adenocarcinoma, was used. Considering the similarity between the pathological and behavioral processes of the Ehrlich tumor and those related to humans, it is commonly employed to study immunological and tumorigenic processes and to investigate the anticancer action of new drugs (Rolim et al., 2017; Feitosa et al., 2021). After 7 days of treatment, ACS-AZ induced a decrease in both evaluated parameters (tumor volume and cell viability). At the dose of 50 mg/kg, ACS-AZ presented the highest antitumor effect. Therefore, this dose was chosen to study the toxicological, antiangiogenic, and immunomodulatory actions, as well as the modulation of oxidative stress in the EAC model. Our findings are consistent with literature reports which have shown the antitumor potential of other acridine derivatives, including alkylamino-acridine derivatives (Cholewiński et al., 2011), spiro-acridines (Silva et al., 2019; Duarte et al., 2020), and 9-aminoacridine derivatives (Belmont and Dorange, 2008; Mangueira et al., 2017; Kozurkova et al., 2020; Kumar et al., 2013; Rupar et al., 2020; El-Sayed et al., 2022).

Cancer development and tissue invasion are directly related to the imbalance of the molecular pathways that regulate the generation of new blood vessels, a process known as angiogenesis. Additionally, angiogenesis is also necessary for the metastasis process. It makes sustained angiogenesis an important hallmark of cancer (Yang et al., 2014; Lopatina et al., 2019; Al-Ostoot et al., 2021). In this context, angiogenesis inhibitors are considered one of the significant cancer treatment strategies since they act to target the signals that support the tumor’s blood supply rather than directly killing tumor cells (Al-Ostoot et al., 2021). In this study, ACS-AZ reduced the peritumoral vascular microdensity, indicating an antiangiogenic action, similarly to other acridine (Duarte et al., 2020; Silva et al., 2020) and 9-aminoacridine derivatives (Mangueira et al., 2017; Das and Kundu, 2021). Therefore, we suggested ACS-AZ as an active compound in the arsenal of acridine derivatives with antiangiogenic action.

The tumor microenvironment maintains tumor-associated neovascularization active by providing an excessive number of pro-angiogenic factors, including ROS, NO, interleukins, and chemokines (Yin et al., 2017). Cytokines are also involved in tumor inflammation, playing a crucial role in angiogenesis and the composition of tumor stroma (Tecchio and Cassatella, 2014; Poeta et al., 2019). Here, we quantified IL-1β, TNF-α, CCL2, IL-4, and IL-10 levels, aiming to identify the potential immunomodulatory of ACS-AZ related to its antitumor activity. Additionally, ROS and NO levels were also evaluated.

The decrease in IL-1β levels induced by ACS-AZ suggests immune modulation. The effects of IL-1β on angiogenesis in the tumor microenvironment have been described. IL-1β signaling promotes angiogenesis by activating JNK or p38 mitogen-activated protein kinase (MAPK) and nuclear factor- κB (NF-κB) signaling, in addition to increasing the expression of VEGF and its receptors in endothelial cells (Guo et al., 2016; Hajek et al., 2018; Jiang et al., 2020). This effect may explain the antiangiogenic action of ACS-AZ. Furthermore, the reduction of IL-1β can be directly related to ROS reduction, also induced by treatment with ACS-AZ, since ROS can directly affect the tumor microenvironment by releasing inflammatory cytokines, among them IL-1β (Gu et al., 2018).

The reduction of CCL2 may also contribute to the antiangiogenic action of ACS-AZ considering its pro-angiogenic feature (Izhak et al., 2012; Liu et al., 2018). This effect is associated with both direct angiogenic action on endothelial cells and an upregulation of angiogenesis-inducing factors such as VEGF (Lim et al., 2016). Furthermore, the literature reports a correlation between increased ROS levels and high CCL2 expression (Li et al., 2014), which allows us to infer that ACS-AZ reduces CCL2 levels while also reducing ROS.

TNF-α is another factor that modulates the tumor microenviroment. Depending on the cellular context, it can have both pro- and antitumor effects by influencing several cell processes, such as angiogenesis, inflammation, immunity, and apoptosis (Ham et al., 2016). Similarly, regarding angiogenesis in cancer, TNF-α can also induce both antiangiogenic and proangiogenic actions in different tumors (Jiang et al., 2020). In this study, ACS-AZ increased TNF-α levels to induce its antitumor effect. By activating different signaling pathways (NF-κB, MAPK, and JAK-STAT), TNF-α induces cell death and exerts potent anticancer action. In addition to its direct toxic effect on tumor cells, the antitumor activity of TNF-α has also been associated with indirect effects of damage to the micro- and macro-vasculature in tumor and stimulation of immune responses. By now, studies have been carried out to evaluate the anticancer efficacy of TNF-α in several tumor types; however, despite the promising anticancer potential, in clinical trials, TNF-α has not been a very successful anticancer agent, mainly because of its severe dose-limiting toxicity after systemic administration (Ham et al., 2016; Shen et al., 2018). Our results showed that ACS-AZ modulates the immune system by increasing TNF-α levels to potentiate antitumor immunity, without inducing significant toxicity on mice bearing Ehrlich tumor.

Finally, ACS-AZ also positively regulated IL-4, which is a cytokine with broad pleiotropic actions on multiple lineages (Mirlekar, 2022). IL-4 is an important cytokine of tumor immunity, detected at high levels in several types of primary and metastatic cancers. On the other hand, literature has shown that together with IL-1β or TGF-β, IL-4 can induce the differentiation of antitumor Th9 cells, inhibiting tumor growth (Xue et al., 2019; Keegan et al., 2021). Additionally, IL-4-induced natural killer cell antitumor activity has also been described in the literature (Vuletić et al., 2020).

In addition to the modulation of cytokines and chemokines, we observed that ACS-AZ exerts antioxidant activity. As ROS is essential for all carcinogenesis stages, tumor cells have higher levels of ROS than normal cells. In this sense, elevated levels of ROS induce DNA and protein damage, and alter signaling pathways and metabolic processes, favoring cell proliferation and tumor growth (Moloney and Cotter, 2018; Fukai and Ushio-Fukai, 2020). Additionally, ROS is known for its angiogenic action by modulating VEGF signaling, different transcription factors, and genes related to angiogenesis, such as NF-κB, p54, and matrix metalloproteinases (MMPs) (Galadari et al., 2017; Huang and Nan, 2019). The results obtained for ACS-AZ indicate that this acridine compound exerts antiproliferative and antiangiogenic actions by reducing ROS levels.

Similarly, continuous exposure to moderate/high concentrations of nitric oxide (NO) stimulates neoplastic transformation and tumor initiation. These effects are related to apoptosis evasion, increased inflammation and proliferation, and immune and chemotherapy resistance (Basudhar et al., 2017; Davila-Gonzalez et al., 2017; Wang et al., 2018). In our study, ACS-AZ reduced the nitrite concentration, a NO indicator. It is worth noting that NO is an important signal in the process of VEGF-induced angiogenesis, increasing blood flow and promoting metastasis (Avtandilyan et al., 2018). Furthermore, other angiogenic factors are stimulated by NO, such as EGFR (epidermal growth factor receptor), angiopoietin-2, and estrogen (Ridnour et al., 2005; Tae et al., 2017). Hence, we can suggest that ACS-AZ has an antiangiogenic action that is also associated with NO reduction.

The high toxicity of antineoplastic drugs is a relevant problem related to drug therapy against cancer. For this reason, we carried out a toxicological evaluation to assess ACS-AZ’s ability to damage vital parameters of mice bearing Ehrlich tumor after seven-day treatment. Of the general evaluated parameters, ACS-AZ reduced only water consumption; however, no changes were observed for feed consumption or body weight. Similarly, no changes were recorded in hematological parameters after ACS-AZ treatment, indicating that this compound does not promote myelosuppression, which is associated with several chemotherapeutic drugs. Unlike ACS-AZ, 5-FU showed hematological toxicity as most anticancer agents (Steger et al., 2012). The biochemical parameters are indicative of renal (creatinine and urea) or liver (AST and ALT) toxicity. However, ACS-AZ did not alter these parameters, suggesting pharmacological safety. Histological analysis was performed to evaluate ACS-AZ toxicity at the tissue level. Histopathological analysis of animals’ kidneys confirms the biochemical tests of renal function, suggesting no renal toxicity. For liver analysis, we detected liver damage even in the control EAC-bearing mice. Previous data have shown that Ehrlich’s tumor itself can cause oxidative stress and liver toxicity (Tousson et al., 2020). These data together point to ACS-AZ’s low toxicity after a 7-day repeat-dose assay.

Therefore, once the acute toxicity and toxicity profile at the therapeutic dose of ACS-AZ (50 mg/kg) have been determined, further studies should be performed to investigate effects on a broad variety of potential targets of toxicity after repeated exposure over a relatively limited period of time (normally 28 or 90 days) in healthy animals. The toxicity endpoints include the effects on the nervous, immune, and endocrine systems. Taken together, all of the results (acute, at the therapeutic dose, and repeated dose tests) should be used for hazard identification and risk assessment.

The pharmacological potential of acridine compounds is not restricted to anticancer action. Acridines are considered privileged scaffolds in drug discovery for protozoan infection and Alzheimer’s disease (Makhaeva et al., 2017). Additionally, analgesic property has also been described for acridine derivatives (Llama et al., 1989; Sondhi et al., 2004; Chandra et al., 2010). Then, we tested ACS-AZ for antinociceptive activity using at least two different experimental models.

First, motor coordination was assessed by the rota-rod test (Fajemiroye et al., 2018). The rota-rod test is important to ensure that the drug’s antinociceptive effects are not due to neurotoxicity, depression, or non-specific relaxation of the Central Nervous System (CNS), avoiding false-positive results (Quintans-Júnior et al., 2010; Braga et al., 2021). Our results showed no changes in motor coordination in mice after ACS-AZ treatment; thus, ACZ-AZ activity in the nociception tests cannot be attributed to myorelaxant or CNS sedative effects.

The use of various models, including chemical and/or thermal stimuli, is essential to detect the antinociceptive activity of a drug since different stimuli mimic distinct types of pain and exhibit the antinociceptive nature of a drug (Bergerot et al., 2006). Here, we used two nociception models to evaluate the antinociceptive activity of ACS-AZ: the formalin test (chemical stimuli) and the hot plate test (thermal stimuli). In addition, we explored the role of the opioid system in the antinociceptive effect.

Our data clearly showed that ACS-AZ reduced the nociceptive activity in the formalin test in the first and second phases, suggesting action in both neurogenic pain and inflammatory processes. The formalin test allows the screening of analgesic and anti-inflammatory drugs. The first phase (neurogenic) begins immediately after the intraplantar injection of formalin and is associated with the stimulation of nociceptors (usually C fibers) by glutamate, histamine, substance P, and nitric oxide. The second phase, related to local inflammation, is more continual and durable and involves the release of pro-inflammatory agents (TNF-α, interleukins, bradykinin, prostaglandins, and serotonin) (Hunskaar and Hole, 1987; Silva et al., 2017; Andrade et al., 2020). In general, analgesic agents with central action inhibit both phases of the formalin test, such as opioid analgesics, while peripheral analgesic drugs suppress only the second phase (Aragão Neto et al., 2019).

These findings for ACS-AZ are similar to the morphine analgesic profile, an opioid with central action characterized as the prototype of the group. Generally, opiates activate three opioid receptors (δ, κ, and μ), which belong to the family of G_i_/G_o_ protein-coupled receptors. These GPCRs trigger the opening of a specific type of potassium channel (GIRK’s) that inhibits voltage-gated calcium channels (VGCC’s), leading to a reduction in neuronal excitability in the CNS to relieve pain. Despite the powerful analgesic effect of morphine, this drug induces severe reactions, including tolerance, respiratory depression, and physical and psychological dependence (Yamanaka and Sadikot, 2013; Esfandyari et al., 2018). Given this, the pharmaceutical industry is committed to developing new drugs with therapeutic activity similar to morphine but with reduced side effects, which are considered limiting factors to their use (Hess et al., 2010; Vilella, 2018).

Through the antinociceptive investigation, we evaluated the central analgesic effects of ACS-AZ through the hot plate test, a method based on thermal stimulation associated with central neurotransmission. Thermal stimulation activates nociceptors (non-myelinated C fibers), which transmit information to specific regions in the CNS, producing an organized nociceptive response (Sulaiman et al., 2009; Mekonnen et al., 2010; Singh et al., 2018). For this reason, this experimental model is used to assess the effects of opioid, sedative, and hypnotic drugs (Yeung et al., 1977; Braga et al., 2021). Similarly to morphine, we observed that ACS-AZ increased the time latency between the contact of the animal’s hind paw with the hot plate and the paw-licking reaction or jumping off the surface. Therefore, our results indicate the contribution of CNS to the action of ACS-AZ. In addition, besides the well-documented central component of analgesic opioid action, the involvement of peripheral opioid receptors in analgesia has also been described (Balogh et al., 2018). Thus, further studies must be carried out to evaluate the peripheral antinociceptive action of ACS-AZ. To date, there are no reports in the literature on the action of acridines in proven models in our nociception study.

To explore these findings, the opioid system’s role in the antinociceptive effect of ACS-AZ was investigated. The neurotransmission opioid system participates in both the modulation and the perception of painful sensations in sites located at the spinal and supraspinal levels (Dash et al., 2015; Esfandyari et al., 2018). Therefore, the animals were again submitted to the formalin test but some groups received pretreatment with the non-selective (naloxone) and μ1-selective (naloxonazine) opioid antagonists. The results showed a reversal of the ACS-AZ antinociceptive effect in both phases of the formalin test and indicated that such an effect is due to the involvement of the opioid pathway, especially of the μ1-opioid receptor.

As a conclusion, the present study showed that ACS-AZ has significant *in vivo* antitumor activity by producing immunomodulation related to antiangiogenic and antioxidant effects and low toxicity. Additionally, we demonstrated that the antinociceptive activity induced by ACS-AZ is mediated by the opioid pathway, especially by μ1-opioid receptors.

## Data Availability

The original contributions presented in the study are included in the article/supplementary materials, further inquiries can be directed to the corresponding author.
